# Prevention of Osteoporosis by Oral Administration of Phytate-Removed and Deamidated Soybean β-Conglycinin

**DOI:** 10.3390/ijms16012117

**Published:** 2015-01-19

**Authors:** Makoto Akao, Ryusuke Abe, Noriko Sato, Atsuko Hasegawa-Tanigome, Hitoshi Kumagai, Hitomi Kumagai

**Affiliations:** 1 Department of Chemistry and Life Science, Nihon University, 1866 Kameino, Fujisawa-shi, Kanagawa 252-0880, Japan; E-Mails: makao@brs.nihon-u.ac.jp (M.A.); r.abe0615@gmail.com (R.A.); foodchem@brs.nihon-u.ac.jp (N.S.); 2 Department of Food Business, Nihon University, 1866 Kameino, Fujisawa-shi, Kanagawa 252-0880, Japan; E-Mail: tanigome.atsuko@nihon-u.ac.jp; 3 Department of Food Science and Nutrition, Kyoritsu Women’s University, 2-2-1 Hitotsubashi, Chiyoda-ku, Tokyo 101-8437, Japan; E-Mail: kumagai@kyoritsu-wu.ac.jp

**Keywords:** phytate removal, deamidation, soybean β-conglycinin, ovariectomy, postmenopausal osteoporosis

## Abstract

Phytate-removed and deamidated soybean β-conglycinin (PrDS) prepared by ion-exchange resins was supplemented to be 4% in the diet administered to ovariectomized rats to investigate its preventive effect on osteoporosis. The apparent calcium absorption rate decreased following ovariectomy and was not replenished by oral administration of phytate-removed soybean β-conglycinin (PrS) or casein. On the other hand, administration of PrDS restored the calcium absorption rate to the same level as the sham group. Markers of bone resorption, such as serum parathyroid hormone (PTH) and urinary deoxypyridinoline (DPD), increased, and the bone mineral density and breaking stress decreased following ovariectomy. However, PrDS supplementation suppressed the changes caused by the decrease in calcium absorption from the small intestine. Therefore, PrDS supplementation shows promise for the prevention of postmenopausal osteoporosis.

## 1. Introduction

Bone is a supporting tissue that maintains its structural integrity and morphology through repetitive resorption and formation. It also acts as a calcium reservoir in the human body and helps to regulate the serum calcium level [1]. However, with age, bone resorption predominates bone formation, eventually leading to osteoporosis [2]. Currently, there are more than 10 million individuals with osteoporosis in Japan, and this number will continue to grow as the population ages. Osteoporosis that develops with age is called involutional osteoporosis and is categorized into two types. Type 1 is postmenopausal osteoporosis, which occurs in women with a depressed estrogen level after menopause. Type 2 is senile osteoporosis, which occurs in both men and women over the age of 70 with a depressed vitamin D level [3]. A lowered estrogen level enhances bone resorption, which causes calcium loss in bones, whereas a lowered vitamin D level reduces the biosynthesis of calcium-binding protein and suppresses calcium absorption in the small intestine. Therefore, in both cases, adequate calcium intake is essential to prevent osteoporosis.

The bioavailability of calcium ranges from 10% to 40%, varying depending on the other substances ingested at the same time. Therefore, it is important to consider not only how much calcium to take, but also what to take with it to enhance its bioavailability. Phytate and oxalate are known to interfere with calcium bioavailability by binding strongly with calcium, thereby making it insoluble [4,5]. Other compounds, such as casein phosphopeptide (CPP), calcium citrate malate (CCM) and poly-γ-glutamate, are known to enhance calcium bioavailability via weak binding, which solubilizes it [6,7,8,9]. Acidic functional groups, such as carboxyl and phosphate groups in their structure, play an important role in enhancing calcium absorption. CPP, CCM and poly-γ-glutamate have been included in foods for specified health uses authorized by the Consumer Affairs Agency of Japan to enhance calcium absorption. However, their applicability is limited because of their characteristic flavor and poor processing properties.

Soybeans have traditionally been used in the Japanese diet and are known to have excellent processing properties. However, soybean proteins contain a high level of phytate, which decreases calcium bioavailability [4,5]. On the other hand, because soybean proteins are rich in glutamine and asparagine residues, the number of carboxyl groups can be increased if the acid amides can be deamidated to produce glutamic acid and aspartic acid residues.

In previous studies, we have shown that a carboxylate-type cation-exchange resin is effective at the conversion of glutamine into glutamic acid and asparagine into aspartic acid [10,11,12] and that soybean proteins deamidated after removal of phytate, a calcium absorption inhibitor, enhanced calcium absorption from the intestine [13]. Moreover, administration of phytate-removed and deamidated soybean proteins (PrDS) has been shown to promote bone formation in young male rats [14,15]. However, the effect of PrDS on involutional osteoporosis has not yet been examined. Therefore, the purpose of the present study was to examine if the oral administration of PrDS is effective at preventing osteoporosis caused by ovariectomy.

## 2. Results and Discussion

### 2.1. Properties of Samples

The degree of deamidation of the PrDS was approximately 20%. The calcium, magnesium and zinc contents of the egg albumin were 0.306 ± 0.046, 0.943 ± 0.010 and 0.002 ± 0.000 mg/g protein; those of phytate-removed soybean β-conglycinin (PrS) were 0.879 ± 0.219, 0.598 ± 0.004 and 0.007 ± 0.000 mg/g protein; those of PrDS were 0.125 ± 0.002, 0.405 ± 0.106 and 0.012 ± 0.001 mg/g protein; and those of casein were 0.418 ± 0.008, 0.044 ± 0.001 and 0.034 ± 0.001 mg/g protein, respectively. The contents of these minerals in each sample protein were negligible in the animal diet compared to their contents in the AIN-76 mineral mix.

### 2.2. Mineral Absorption Rate

**Figure 1:**
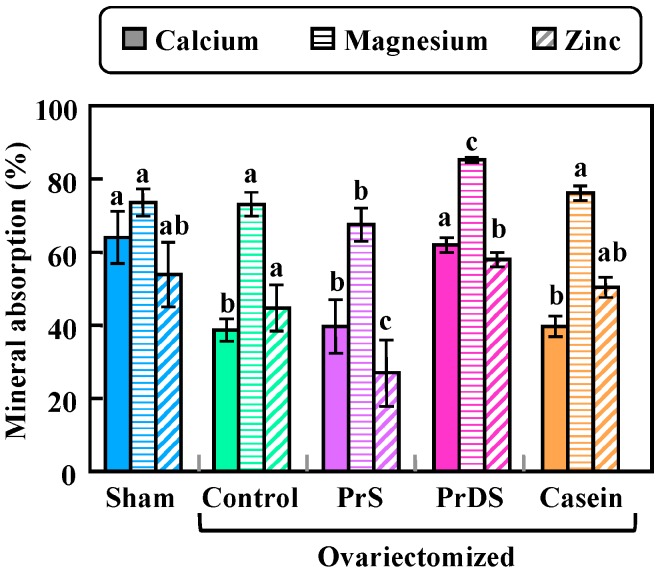
Apparent mineral absorption rate in sham-treated and ovariectomized rats fed each experimental diet for a week. Sham, non-ovariectomized and 20% egg albumin intake; control, ovariectomized and 20% egg albumin intake; phytate-removed soybean β-conglycinin (PrS), ovariectomized and 4% PrS + 16% egg albumin intake; Phytate-removed and deamidated soybean β-conglycinin (PrDS), ovariectomized and 4% PrDS + 16% egg albumin intake; casein, ovariectomized and 4% casein + 16% egg albumin intake. Each value shows the mean for six rats with the standard error (SE). Absorption values for the same mineral indicated by different letters are significantly different at *p* < 0.05.

At the beginning of the mineral balance study, no individual differences were observed among the sample groups (data not shown). After one week of feeding of each experimental diet, the calcium absorption rate was reduced in the ovariectomized groups, but a similar reduction was not observed in the group fed PrDS. The calcium absorption rate of the PrDS group was significantly higher than that of the PrS and casein groups and was almost equal to that of the sham group (Figure 1; sham: 63.73% ± 6.96%; control: 38.46% ± 3.01%; PrS: 39.48% ± 7.27%; PrDS: 61.80% ± 2.08%; casein: 39.53% ± 2.77%). These results indicate that the increase in carboxyl groups by deamidation of glutamine and asparagine residues was effective enough to enhance calcium bioavailability via their weak binding with calcium ions in the gut. Numerous studies have reported that estrogen deficiency brought on by ovariectomy causes a reduction in intestinal calcium absorption [16,17,18,19]. The same phenomenon was observed in this study. The absorption rates of magnesium (Figure 1; sham: 73.06% ± 3.80%; control: 72.56% ± 3.38%; PrS: 67.14% ± 4.66%; PrDS: 84.63% ± 0.72%; casein, 75.86% ± 2.01%) and zinc (Figure 1; sham: 53.72% ± 8.86%; control: 44.68% ± 6.46%; PrS: 26.75% ± 9.03%; PrDS: 57.52% ± 2.05%; casein: 49.89% ± 2.86%) were not decreased by ovariectomy, but in comparison to the non-ovariectomized sham group, the PrS group showed significantly lower and the PrDS group showed higher values. Approximately two-thirds of all magnesium in bone is in the form of hydroxyapatite crystals, and the magnesium content is associated with the qualitative changes in hydroxyapatite [20]. Moreover, magnesium supplementation has been reported to increase bone strength in ovariectomized rats [21]. Therefore, the increased magnesium absorption rate caused by PrDS intake may also strengthen bone. According to our previous *in situ* study, which examined the calcium absorption rate from the small intestine [15], PrDS enhanced the calcium absorption rate more strongly than casein, whereas PrS did not have an enhancing effect. A similar trend was observed in the present work: PrDS was more effective than casein, and PrS was ineffective. Although CPP is known to enhance calcium absorption from the small intestine, the amount of CPP produced from casein in the gut would not have been sufficient to have an effect. From these findings, daily intake of a diet containing 4% PrDS was expected to increase bone mineral density and strength. As an intake of 4% PrDS is almost equivalent to that of 4 g PrDS/kg body weight, daily intake of 200–250 g of PrDS per person would be necessary to have the same effect.

### 2.3. Levels of Bone Resorption Markers

**Figure 2:**
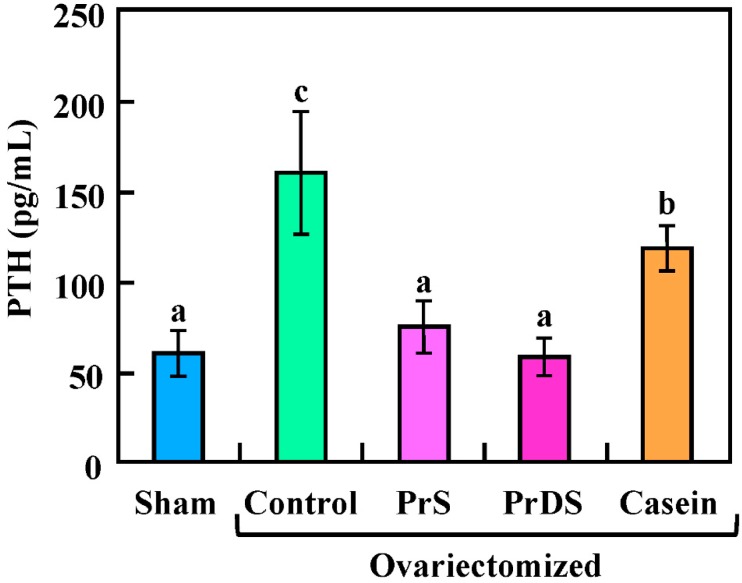
Serum parathyroid hormone (PTH) level in sham-treated and ovariectomized rats fed each experimental diet for eight weeks. Sham, non-ovariectomized and 20% egg albumin intake; control, ovariectomized and 20% egg albumin intake; PrS, ovariectomized and 4% PrS + 16% egg albumin intake; PrDS, ovariectomized and 4% PrDS + 16% egg albumin intake; casein, ovariectomized and 4% casein + 16% egg albumin intake. Values show the means for six rats with the SE. Values indicated by different letters are significantly different at *p* < 0.05.

The serum parathyroid hormone (PTH) level is shown in Figure 2 (sham: 60.49 ± 14.57 pg/mL; control: 159.89 ± 38.05 pg/mL; PrS: 75.14 ± 14.90 pg/mL; PrDS: 59.18 ± 11.68 pg/mL; casein: 119.3 ± 17.23 pg/mL). When the calcium concentration in the blood decreases, PTH is secreted from the parathyroid gland to increase bone resorption [22]. As the level of secreted estrogen decreases due to the onset of menopause, bone resorption is accelerated by increasing the PTH level [23]; estrogen deficiency brought on by ovariectomy also causes an increase in the PTH level [24]. A similar pattern was observed in the present study: the PTH level was significantly higher in the control group than in the sham group. On the other hand, the PTH level of the PrDS group was almost identical to that of the sham group, suggesting sufficient calcium absorption from the small intestine. Suppression of PTH secretion due to the enhancement of calcium absorption from the small intestine was also observed for young male rats administered PrDS, which led to the promotion of bone formation [25]. Although no enhancing effect of calcium absorption was observed for PrS after one week of feeding (Figure 1), the PTH level of the PrS group was almost comparable to that of the sham group. The calcium bioavailability might have been improved during the intake of PrS for eight weeks.

**Figure 3:**
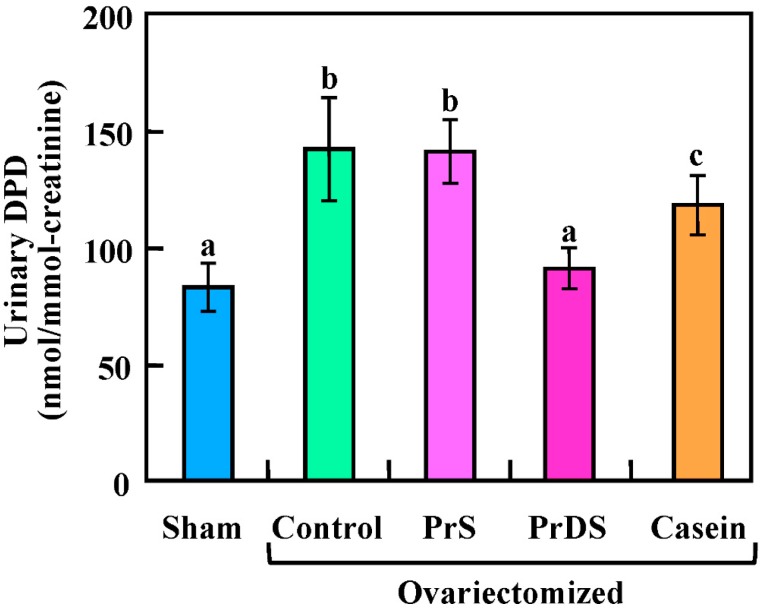
The urinary deoxypyridinoline (DPD) level in sham-treated and ovariectomized rats fed each experimental diet for eight weeks. Urinary DPD was standardized with urinary creatinine. Sham, non-ovariectomized and 20% egg albumin intake; control, ovariectomized and 20% egg albumin intake; PrS, ovariectomized and 4% PrS + 16% egg albumin intake; PrDS, ovariectomized and 4% PrDS + 16% egg albumin intake; casein, ovariectomized and 4% casein + 16% egg albumin intake. Values represent the means for six rats with the SE. Values indicated by different letters are significantly different at *p* < 0.05.

The level of deoxypyridinoline (DPD) is shown in Figure 3 (sham group: 83.31 ± 5.24 nmol/mmol creatinine; control: 142.34 ± 26.7 nmol/mmol creatinine; PrS: 140.82 ± 12.0 nmol/mmol creatinine; PrDS: 91.2 ± 4.04 nmol/mmol creatinine; casein: 118.05 ± 9.64 nmol/mmol creatinine). Type 1 collagen accounts for approximately 90% of the organic matrix of bone and is cross-linked by pyridinoline and DPD, which are released and excreted in the urine during bone resorption and collagen breakdown. Therefore, the presence of DPD in the urine is considered to be a marker of bone resorption [26]. The control, PrS and casein groups showed significantly higher DPD levels than the sham group. However, the DPD level of the PrDS group was almost identical to that of the sham group.

These results suggest that daily intake of 4% PrDS suppresses bone resorption following ovariectomy and may promote the storage of calcium in bone, which can lead to increased bone mineral density and strength. The suppressing effect of casein on bone resorption was lower than that of PrDS, probably because of its lower calcium absorption rate.

### 2.4. Bone Mineral Density

**Figure 4:**
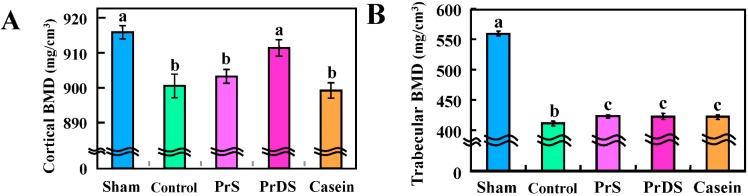

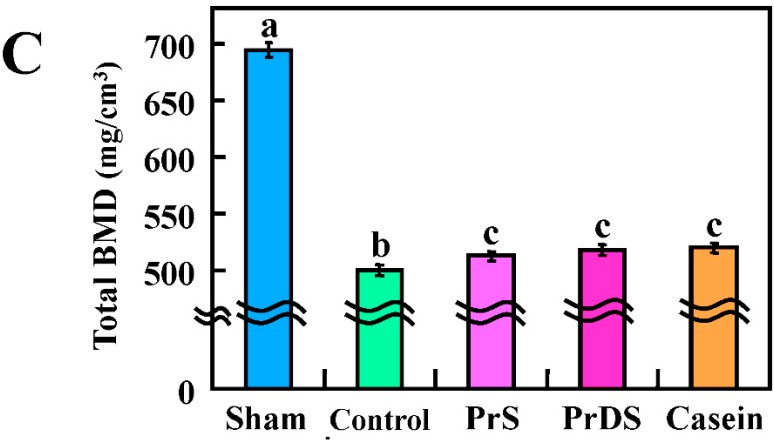
Bone mineral density (BMD) at 4 mm from the endpoint of femurs in sham-treated and ovariectomized rats fed each experimental diet for eight weeks. (**A**) Cortical, (**B**) trabecular and (**C**) total BMD. Sham, non-ovariectomized and 20% egg albumin intake; control, ovariectomized and 20% egg albumin intake; PrS, ovariectomized and 4% PrS + 16% egg albumin intake; PrDS, ovariectomized and 4% PrDS + 16% egg albumin intake; casein, ovariectomized and 4% casein + 16% egg albumin intake. Values represent the means for six rats with the SE. Values indicated by different letters for the same BMD are significantly different at *p* < 0.05.

Femur bone mineral density (BMD) is shown in Figure 4. Cortical BMD (sham: 915.04 ± 1.79 mg/cm^3^; control: 901.17 ± 3.01 mg/cm^3^; PrS: 903.68 ± 1.85 mg/cm^3^; PrDS: 911.01 ± 2.11 mg/cm^3^; casein: 899.99 ± 1.98 mg/cm^3^) and trabecular BMD (sham: 557.63 ± 3.52 mg/cm^3^; control: 421.37 ± 3.65 mg/cm^3^; PrS: 432.28 ± 2.86 mg/cm^3^; PrDS: 432.17 ± 4.52 mg/cm^3^; casein: 431.2 ± 3.47 mg/cm^3^) were individually evaluated by peripheral quantitative computed tomography (pQCT) [27,28] along with total BMD (sham: 685.52 ± 6.42 mg/cm^3^; control: 500.19 ± 4.40 mg/cm^3^; PrS: 512.47 ± 3.96 mg/cm^3^; PrDS: 516.88 ± 4.87 mg/cm^3^; casein: 518.81 ± 4.03 mg/cm^3^). Since trabecular bone has a greater surface area than cortical bone, the remodeling rate of trabecular bone is thought to be faster than that of cortical bone [29,30], and approximately 10% of cortical bone is remodeled annually [31]. In addition, the effects of estrogen deficiency are expected to be seen first in trabecular bone [32]. Therefore, femur BMD was measured at 4 mm from the endplate where longitudinal growth occurs and trabecular bone is dominant. As expected, trabecular BMD decreased to 75.6% (=(421.37 mg/cm^3^)/(557.63 mg/cm^3^) × 100) following ovariectomy, while cortical BMD decreased only to 98.5% (=(901.17 mg/cm^3^)/(915.04 mg/cm^3^) × 100). However, a noticeable difference was observed between groups, even in cortical BMD. The cortical BMD of the PrDS group was significantly higher than that of any other ovariectomized groups and almost identical to that of the sham group (Figure 4A). The reduction occurring in trabecular BMD following ovariectomy was slightly restored in the PrS, PrDS and casein groups (Figure 4B). Because the remodeling rate of cortical bone is slow, the BMD of the PrDS group may have been retained by the suppression of bone resorption rather than by enhancement of bone formation. The cortical bone constitutes the hard outer layer and is composed of compact tissues, which account for 80% of the total bone mass. Therefore, cortical BMD affects bone strength, and the PrDS group was expected to have greater bone strength than the other ovariectomized groups.

### 2.5. Bone Bending Stress

**Figure 5:**
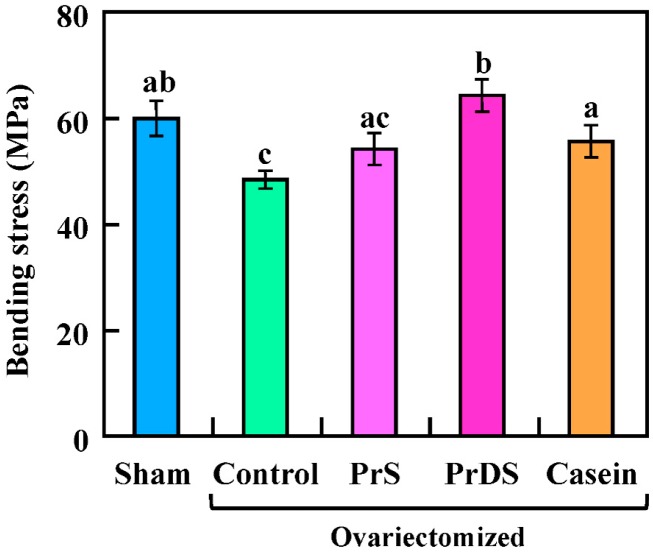
Bending stress of femurs in sham-treated and ovariectomized rats fed each experimental diet for eight weeks. Sham, non-ovariectomized and 20% egg albumin intake; control, ovariectomized and 20% egg albumin intake; PrS, ovariectomized and 4% PrS + 16% egg albumin intake; PrDS, ovariectomized and 4% PrDS + 16% egg albumin intake; casein, ovariectomized and 4% casein + 16% egg albumin intake. Values represent the means for six rats with the SE. Values indicated by different letters are significantly different at *p* < 0.05.

The bending stress of femurs measured using the three-point bending test is shown in Figure 5 (sham: 59.71 ± 3.38 MPa; control: 48.57 ± 1.51 MPa; PrS: 54.34 ± 2.98 MPa; PrDS: 64.01 ± 3.00 MPa; casein: 55.52 ± 3.08 MPa). Ovariectomy reduced the amount of stress required to break the bone, but it was restored in the PrDS group, which showed a significantly higher bending stress tolerance than any other ovariectomized groups. These results are concordant with those for mineral absorption rate, bone-resorption markers and BMD. Thus, PrDS supplementation shows promise for preventing osteoporosis by enhancing mineral absorption from the small intestine, suppressing bone resorption and enhancing BMD.

## 3. Experimental Section

### 3.1. Materials

Phytase-treated soybean β-conglycinin (Lipoff-700™, Fuji Oil Co., Ltd., Osaka, Japan) was used in this study, because phytate interferes with calcium absorption from the small intestine. The phytate content in this was confirmed to be negligible through the measurement as phosphorus by inductively-coupled plasma atomic emission spectroscopy (ICP). Ammonium sulfate, 2-mercaptoethanol, potassium carbonate, sodium hypochlorite and phenol were purchased from Nacalai Tesque, Inc. (Tokyo, Japan). Casein was obtained from Wako Pure Chemical Industries, Ltd. (Osaka, Japan), and the cation-exchange resin used was Amberlite IRC 76 obtained from Organo Co., Tokyo, Japan. All other chemicals were of reagent grade.

### 3.2. Removal of Isoflavone

Isoflavone was removed from phytase-treated soybean β-conglycinin in order to eliminate its effect on bone resorption. It was washed with a 10-times volume of 70% ethanol and centrifuged at 1100× *g* for 10 min to obtain a precipitate. This procedure was repeated 4 times. The isoflavone was effectively removed from phytase-treated soybean β-conglycinin, which was confirmed through the measurement by the Folin–Ciocalteu method. The isoflavone and phytate-free β-conglycinin was used as PrS.

### 3.3. Deamidation of Phytate-Removed Soybean β-Conglycinin (PrS)

PrS was deamidated by using a carboxylate type cation-exchange resin, as described previously [13], with some modifications. A glass column was packed with 600 mL of the cation-exchange resins, successively washed with 1 N NaOH, deionized water, 1 N HCl, deionized water, 0.1 N NaOH, deionized water and equilibrated with 0.01 N NaOH prior to use. Then, 20 mL of 6% PrS were applied to the column and eluted with 0.01 N NaOH at a flow rate of 3 mL/min. The PrDS was obtained in the eluent, followed by dialysis against water and lyophilization.

The degree of deamidation was determined as the ratio of the amount of acid amide removed by treatment with the cation-exchange resin to the total acid amide in PrS [13]. Briefly, the acid amides in PrS and PrDS were both completely deamidated by heating in an HCl solution, and the amount of ammonia produced was measured by Conway’s micro-diffusion method [33] and the indophenol method [34]. Then, the amount of acid amide deamidated was calculated by subtracting the amount of ammonia produced from the PrDS from that produced from the PrS.

### 3.4. Breeding Conditions

For acclimation to the animal house environment, 6-week-old female Wistar rats (Clea Japan, Inc., Tokyo, Japan) were kept at 23 °C with a 12-h light/12-h dark cycle and fed a standard diet based on the AIN-76 formulation (Oriental Yeast Co., Ltd., Tokyo, Japan) containing 20% egg albumin for 7 days. Then, the rats were divided into 5 groups; 1, sham-operated (sham group); 2, ovariectomized (control group); 3, ovariectomized + PrS (PrS group); 4, ovariectomized + PrDS (PrDS group); 5, ovariectomized + casein (casein group); all were fed a restricted diet of 20 g/day for 8 weeks. The control/sham groups were fed the standard diet containing 20% egg albumin, while the other groups (PrS, PrDS and casein groups) were fed a diet containing 16% of the egg albumin and 4% of each experimental protein (PrS, PrDS or casein). The composition of each diet is summarized in Table 1.

After 8 weeks of the experimental period, the rats were fasted for 18 hours, and blood samples were collected from the jugular vein. Sera were obtained by centrifugation of the blood at 3000× *g* and 20 °C for 15 min. The femurs were excised from the rats and stored in 70% ethanol after removal of muscles and connective tissues. The animal experiments were performed in accordance with the Guidelines for Animal Experiments of the College of Bioresource Sciences, Nihon University.

**Table 1:** Composition of the experimental diet.

Ingredient	Experimental Group (g/kg Diet)			
	Control and Sham	PrS	PrDS	Casein
Egg albumin	200	160	160	160
PrS		40		
PrDS			40	
Casein				40
DL-methionine	3	3	3	3
Corn starch	150	150	150	150
Sucrose	500	500	500	500
Cellulose powder	50	50	50	50
Corn oil	50	50	50	50
AIN-76 mineral mix	35	35	35	35
AIN-76 vitamin mix	10	10	10	10
Choline bitartrate	2	2	2	2

### 3.5. Apparent Mineral Absorption Rate

Feces were collected using metabolic cages for 24 h on the first and 7th days of the experimental period, and their weights were measured after being dried under vacuum. Minerals (calcium, magnesium and zinc) were extracted from 100 mg of dried feces by wet-ashing. The calcium, magnesium and zinc concentrations of the feces were measured by inductively-coupled plasma atomic emission spectroscopy (ICP, SPS1700R, Seiko Instruments Inc., Tokyo, Japan). The apparent rate of mineral absorption, A (%), was calculated using the equation below.

*A* (%) = (*I* − *E*)/*I* × 100 (1)

where *I* (mg/day) is the mineral intake from the experimental diet and *E* (mg/day) is the mineral excretion in feces.

### 3.6. Assays of Serum Parathyroid Hormone and Urinary Deoxypyridinoline

The serum PTH level was measured as an indicator of the calcium absorption level from the small intestine using a PTH rat enzyme-linked immunosorbent assay (ELISA) system (GE Healthcare Bio-Sciences K.K., Tokyo, Japan).

The urinary DPD level was measured as an indicator of bone resorption using a DPD ELISA kit (DS Pharma Biomedical Co., Ltd., Osaka, Japan). DPD was standardized with urinary creatinine, which was measured using a creatinine test kit (Wako Pure Chemical Industries, Ltd., Osaka, Japan).

### 3.7. Measurement of Bone Mineral Density

Femur bone mineral density (BMD) was measured based on loop analysis using peripheral quantitative computed tomography (pQCT, XCT Research SA, Stratec Medizintechnik GmbH, Pforzheim, Germany). Femurs were fixed in a tube and scanned at a slice thickness of 0.75 mm and a voxel size of 0.1 mm with a scan speed of 10 mm/s. The scan line was adjusted to 4 mm from the distal end of the femur using scout view. Trabecular and cortical bones were defined and analyzed using contour Mode 1 (threshold: 267 mg/cm^3^), peel Mode 2 (threshold: 690 mg/cm^3^) and separation Mode 1 (threshold: 750 mg/cm^3^). It was assumed that a density of 267–690 mg/cm^3^ indicated trabecular bone and a density greater than 750 mg/cm^3^ indicated cortical bone.

### 3.8. Measurement of Mechanical Bone Strength

Mechanical bone strength was evaluated by the three-point bending test, using a mechanical testing machine (INSTRON 5567, Instron Corp., Norwood, MA, USA). The bones were positioned horizontally and rested on two support plates that were 10 mm apart. The pressing force was directed vertically to the midpoint of the bone with a plunger speed of 2 mm/min. The breaking force was defined as the bending load at failure. The cross-section of the femur was approximated as an oval. The maximum bending stress, σ (Pa), was calculated using the equation below.

σ = *F L*/π *r*_a_^2^* r*_b_ (2)

where *F* (N) is the maximum load at failure, *L* (m) is the distance between the support and *r*_a_ (m) and *r*_b_ (m) are the cross-sectional radii at the midpoint of the femur.

## 4. Conclusions

PrDS prepared by deamidation of PrS contained high quantities of aspartic and glutamic acid residues, which can bind to cations, such as calcium, magnesium and zinc. PrDS would interact with these minerals and help with solubilizing them in the gut, leading to the enhancement of their absorption from the small intestine. The enhanced mineral absorption reduced bone resorption by suppressing the secretion of PTH from the parathyroid gland and the breakdown of collagen in bone, as shown by the reduction in the DPD level. The reduction in bone resorption enhanced BMD and strengthened bone. Therefore, deamidation of soybean protein after removal of phytate, a calcium absorption inhibitor, is effective at preventing osteoporosis caused by estrogen deficiency.
